# The Pathogenicity of COVID-19 Is Independent of Increasing Altitude: The Case of Colombia

**DOI:** 10.4269/ajtmh.20-1465

**Published:** 2021-02

**Authors:** Valeria Janice Valverde-Bruffau, Laura Cárdenas, Gustavo F. Gonzales

**Affiliations:** High Altitude Research Institute; Laboratories of Investigation and Development (LID); Department of Biological and Physiological Sciences; Faculty of Sciences and Philosophy; Universidad Peruana Cayetano Heredia; Lima, Peru; E-mail: valeria.valverde.b@upch.pe; Department of Biological and Physiological Sciences; Faculty of Sciences and Philosophy; Universidad Peruana Cayetano Heredia; Lima, Peru; E-mail: laura.cardenas.z@upch.pe; High Altitude Research Institute; Laboratories of Investigation and Development (LID); Department of Biological and Physiological Sciences; Faculty of Sciences and Philosophy; Universidad Peruana Cayetano Heredia; Lima, Peru; E-mail: gustavo.gonzales@upch.pe

Dear Sir,

The recent article “Negative Correlation between Altitude and COVID-19 Pandemic in Colombia: A Preliminary Report” published by Cano-Perez et al.^1^ analyzed data from 70 municipalities in Colombia and concluded that increasing altitude was associated with fewer SARS-2 infections, lower COVID-19 mortality, and lower case fatality rate (CFR).^1^ These results are consistent with studies from the beginning of the pandemic in eight provinces of Bolivia, from Ecuador,^2^ and from 185 provinces in Peru.^3^ This latest study concluded that CFR was unchanged with increasing altitude.^3^

A recent study in 1,636 districts in Peru located between 5 and 4,705 m above sea level assessed 509,521 cases with SARS-CoV-2 infection. A negative correlation between altitude and infections with SARS-CoV-2 was observed; however, further analysis showed that the negative correlation was only observed between 5 and 1,000 m altitude. At altitudes between 1,000 and 4,700 m, there was no significant association between altitude and incidence of infection.^4^ An altitude gradient for protection against SARS-CoV-2 contagion could not be demonstrated.

Regarding the publication from Colombia,^1^ only 70 municipalities from a total of 1,122 were analyzed. This limited sample might produce a bias in the interpretation of the results. Therefore, information from the 1,122 municipalities in Colombia ranging in altitude from 2 to 3,259 m above sea level has been analyzed.

The database that includes population, land area (km^2^), and altitude (m) of the municipalities was obtained from the official website of the municipalities of Colombia, and the data regarding positive and deceased cases were obtained from the official website of the Colombian Ministry of Health. Of the total municipalities, 536 are located from 0 to < 1,000 m altitude, with an area of 932,014 km^2^ and a population of 21,406,305 inhabitants, and 586 municipalities from 1,000 to 3,259 m, with an area of 208,952 km^2^ and a population of 22,761,341 inhabitants. Bogotá, the capital of Colombia, located at 2,630 m above sea level, has an area of 1,623 km^2^ and a population of 7,200,000 inhabitants.

As of November 1, 2020, Colombia has recorded 1,073,123 SARS-CoV-2 infections and 31,312 COVID-19 deaths, with a CFR (deaths/cases × 100) of 2.9%. The municipalities located below 1,000 m altitude have recorded 442,795 SARS-CoV-2 infections and 16,691 COVID-19 deaths, with a CFR of 3.8%, whereas municipalities located from 1,000 to 3,259 m have recorded 632,410 SARS-CoV-2 infections and 14,621 COVID-19 deaths, with a CFR of 2.3%. Data from < 1,000 m altitude and from ≥ 1,000 to 3,259 m were assessed by the Spearman correlation, Pearson correlation, and Poisson regression analysis. Data from Poisson regression analysis were presented as adjusted *R*^2^ with its *P*-value and included the β coefficient and its *P*-value.

The results after Spearman’s analysis showed a significant negative correlation between both SARS-CoV-2 infections and COVID-19 deaths and altitude < 1,000 m (*P* < 0.05), whereas no significant difference was seen for altitudes between 1,000 and 3,259 m (*P* > 0.05). The CFR decreased as the altitude of residence increased for both groups (< 1,000 m and ≥ 1,000 m) (*P* < 0.05) (Table 1).

**Table 1:** Analysis of correlation of the Spearman and Poisson regression analyses of cases of SARS-CoV-2, deaths due to COVID-19, and CFR in relation to altitude (m) with and without adjustment by population density from 1,122 municipalities of Colombia

	Confirmed cases		Deaths		CFR	
	< 1,000	≥ 1,000	< 1,000	≥ 1,000	< 1,000	≥ 1,000
Spearman’s correlation	−0.1132*	0.0049	−0.1658*	0.0173	−0.1244*	−0.1127*
Altitude						
Adjusted *R*^2^	0.0058*	0.0356*	0.0007*	0.0401*	0.0002	0.0043*
β Coefficient	4.7 × 10^−4^*	9.5 × 10^−4^*	1.7 × 10^−4^*	0.001*	−4.4 × 10^−5^	−1.3 × 10^−4^*
Population density						
Adjusted *R*^2^	0.2971*	0.3040*	0.3486*	0.2851*	0.0020*	0.0322*
β Coefficient	4.98 × 10^−7^*	4.23 × 10^−7^*	5.13 × 10^−7^*	4.16 × 10^−7^*	−8.48 × 10^−8^*	−4.93 × 10^−7^*
Density and altitude						
Adjusted *R*^2^	0.3182*	0.3810*	0.3594*	0.3665*	0.0021*	0.0370*
β Coefficient density	5.29 × 10^−7^*	4.93 × 10^−6^*	5.35 × 10^−7^*	4.88 × 10^−7^*	−8.42 × 10^−8^*	−4.94 × 10^−7^*
β Coefficient altitude	9.5 × 10^−4^*	0.001*	6.9 × 10^−4^*	0.001*	−4.2 × 10^−5^	−1.4 × 10^−5^

CFR = case fatality rate. Data are obtained from 1,122 districts of Colombia (https://sig.sispro.gov.co/SituacionCovid/) and data of the altitudes are obtained of official Municipio page (https://www.municipio.com.co/).

* *P* < 0.05. Adjusted *R*^2^ of the regression of Poisson corresponds to each model: first model: altitude; second model: population density; third model: population density and altitude. The coefficient beta of each independent variable is also included.

**Figure 1.**
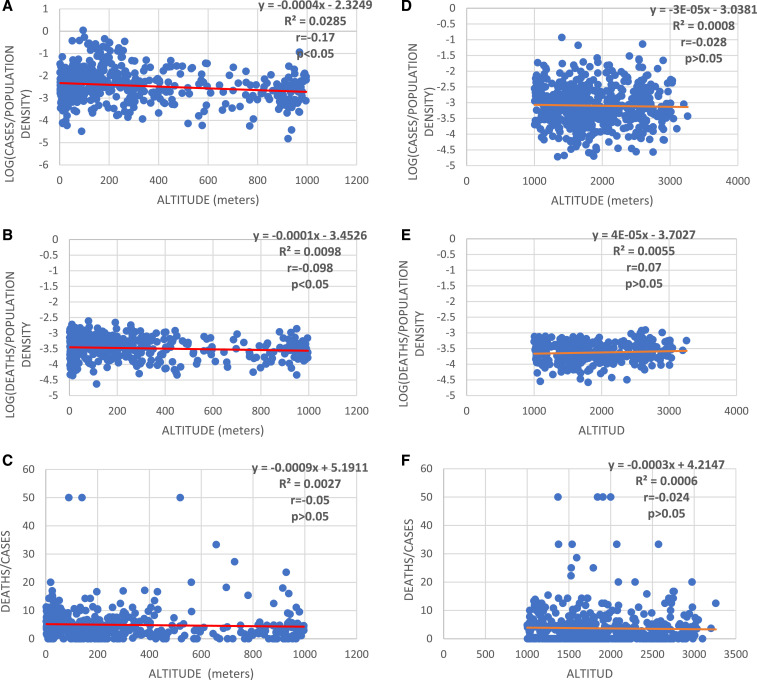
(**A**) Association of positive cases to SARS-CoV-2 adjusted with population density in relation to altitude between 0 and < 1,000 m (*P* < 0.05). (**B**) Association of deaths from COVID-19 adjusted to the population density between 0 and < 1,000 m of altitude (*P* < 0.05). (**C**) Association of CFR (%) with altitude between 0 and < 1,000 m (*P* > 0.05). (**D**) Association of positive cases to SARS-CoV-2 adjusted to the population density between 1,000 and 3,259 m of altitude (*P* > 0.05). (**E**) Association of deaths from COVID-19 adjusted to the population density between 1,000 and 3,259 m of altitude (*P* > 0.05). (**F**) Association of CFR (%) with the altitude between 1,000 and 3,259 m (*P* > 0.05). CFR = case fatality rate. This figure appears in color at www.ajtmh.org.

According to the Pearson correlation, the only significant difference was observed for SARS-CoV-2 infections and COVID-19 deaths at altitudes < 1,000 m. However, the slope was close to zero, and biological significance is likely negligible (Figures 1A–F).

Poisson regression was performed with only altitude in the model, only population density in a second model, and including altitude and population density in a third model. With only altitude in the model, a positive association was observed between infections and deaths in both altitude groups (*P* < 0.05). However, the CFR showed a negative association with altitude only in the group living at between 1,000 and 3,259 m (*P* < 0.05).

When only population density was incorporated in the model, more confirmed infections and deaths occurred as population density increased in both altitudinal groups (*P* < 0.05). The CFR decreased as population density increased in both altitudinal groups (*P* < 0.05).

In the third model, altitude and the population density were associated with dependent variables (infections, deaths, and CFR) for each altitudinal group. Positive associations were observed between infections and deaths in both altitudinal groups (*P* < 0.05), and for β coefficients for density (*P* < 0.05) and altitude (*P* < 0.05) variables. Case fatality rate had a negative association with density (*P* < 0.05) in both groups; however, no significant association was observed between increasing altitude and CFR in both altitudinal groups (*P* > 0.05).

In conclusion, data from Colombia show that there is no altitude gradient that is protective against SARS-CoV-2 infection or COVID-19 mortality.
